# Supplementary material for: Assessment of Lipopeptide Mixtures Produced by Bacillus subtilis as Biocontrol Products against Apple Scab (Venturia inaequalis)
Source: Microorganisms. 2022 Sep 9;10(9):1810. doi: 10.3390/microorganisms10091810 (PMC9501572; doi:10.3390/microorganisms10091810)
Supplement: Supplementary file 1 [file microorganisms-10-01810-s001.zip › microorganisms-1841786-supplementary.pdf]

Supplementary materials

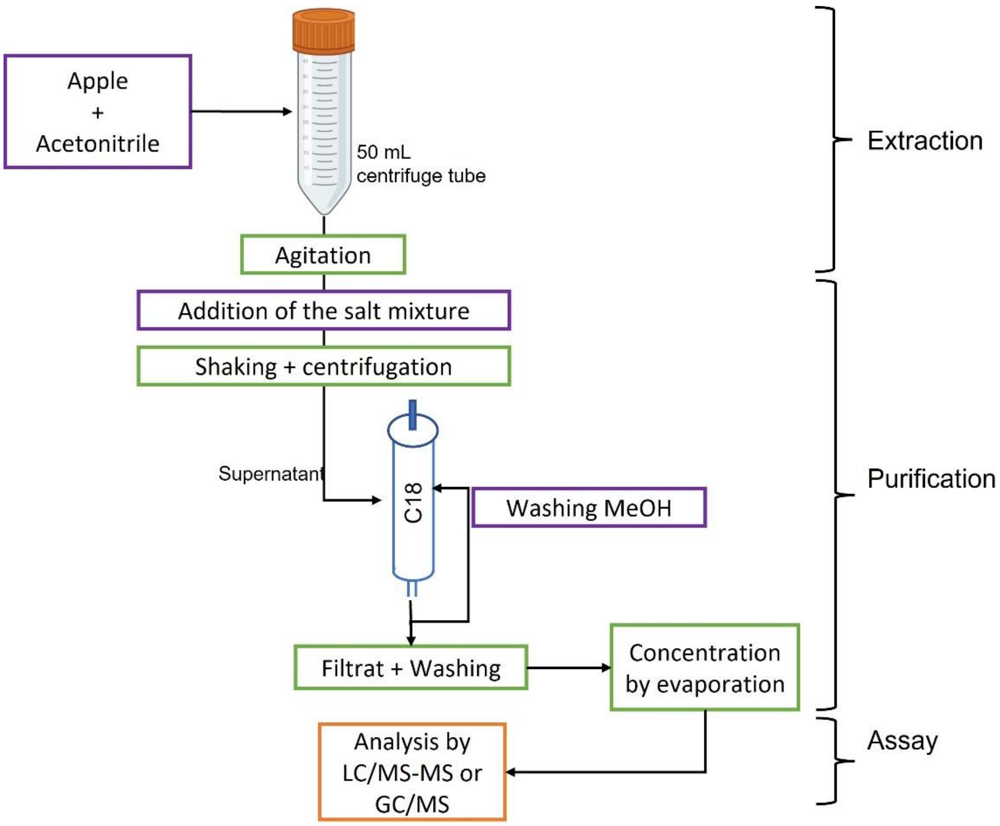

Figure S1. Schematic representation of the QuEChERS method used to evaluate the persistence of the lipopeptides on fruit

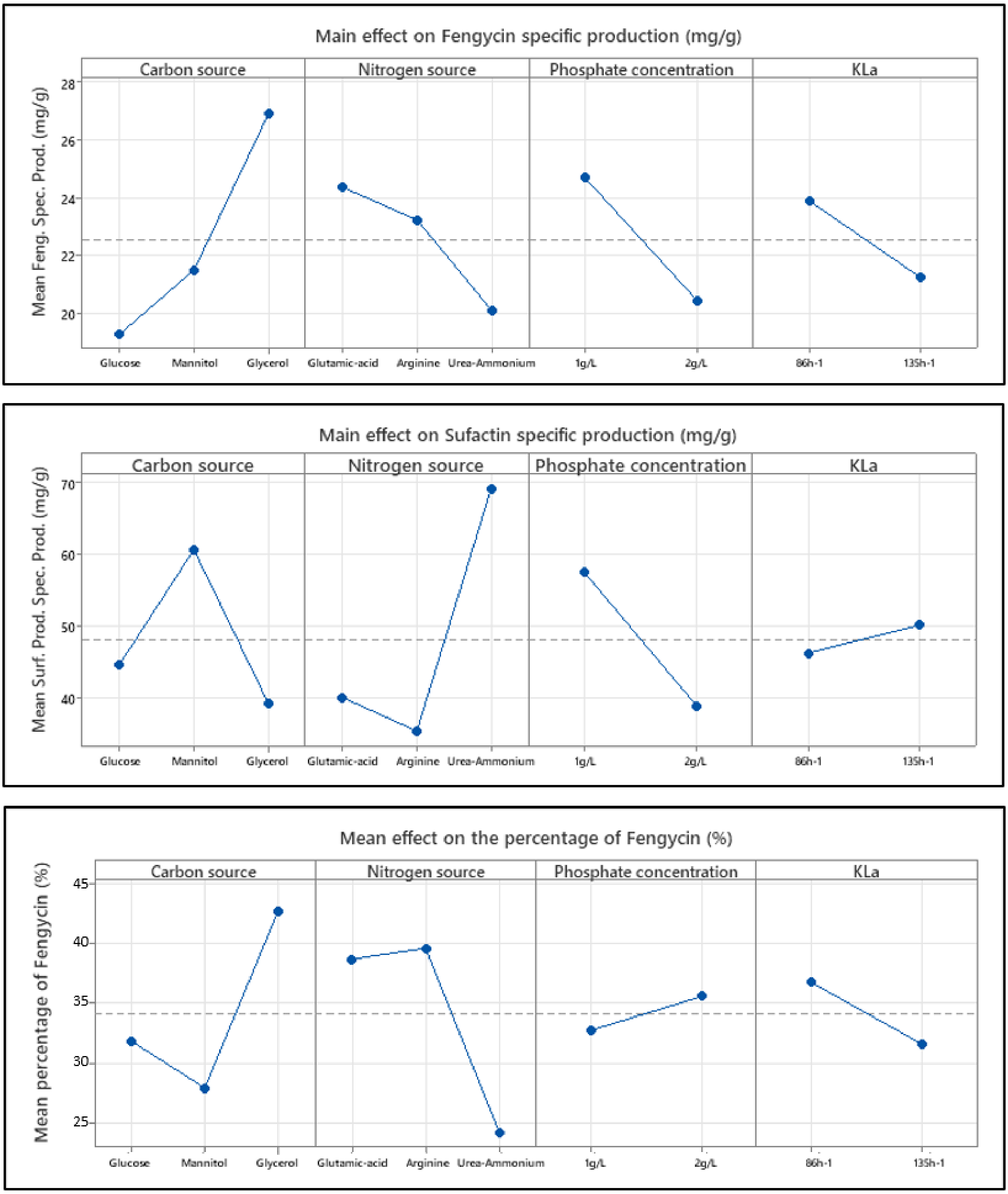

Figure S2. Diagram of the mean effect on fengycin specific production, surfactin specific production and on the percentage of Fengycin produced

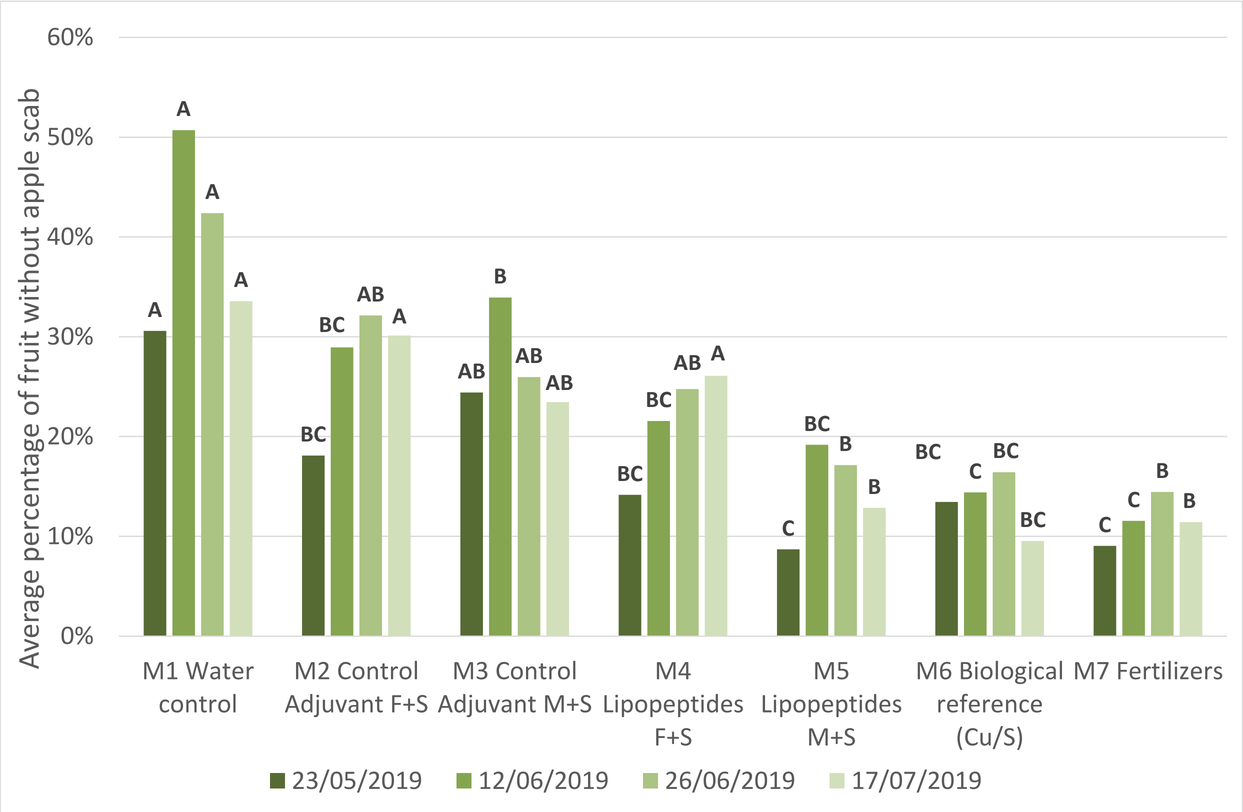

Figure S3. Graphical representation of evolution of the percentage of scab leaves for each modality in 2019. The letters above each histogram (A, B, AB, C, BC) correspond to the statistical group to which the modality belongs after analysis of variance, for each date of notation.

Table S4 : Percentage of spots and effectiveness (on the right) of the different modalities against apple scab on foliage compared to the reference of the trial (M6) in 2019

	M6 Biological reference (Cu/S)	M1 Water control		M2 Control Adjuvant F+S		M3 Control Adjuvant M+S		M4 Lipopeptides F+S		M5 Lipopeptides M+S		M7 Fertilizers	
23/05/2019	13,5%	30,6%	0,0%	18,1%	0,0%	24,4%	0,0%	14,2%	0,0%	8,7%	35,4%	9,1%	32,8%
12/06/2019	14,4%	50,7%	0,0%	28,9%	0,0%	33,9%	0,0%	21,6%	0,0%	19,2%	0,0%	11,6%	19,8%
26/06/2019	16,4%	42,4%	0,0%	32,1%	0,0%	26,0%	0,0%	24,8%	0,0%	17,1%	0,0%	14,4%	12,1%
17/07/2019	9,5%	33,6%	0,0%	30,1%	0,0%	23,5%	0,0%	26,1%	0,0%	12,9%	0,0%	11,4%	0,0%
